# 

**Published:** 1888-04-21

**Authors:** 


### CONTENTS.

**Nurse-Training Schools and Registration** - - 37

**Words of Consolation.** Chapter LXXIV. - - - 38

**The Doctor's Pulpit.**—Constitution Building - - 39

**Scotch Physicians and Surgeons.** By C. G. Furley.—James Syme. - - - - - - - - 40

**The New Great Northern Central Hospital** - - 41

**The Jaeger Revolution** - - - - - 42

**In and Out Among the Hospitals** - - - - 43

**The Land of Golden Day** - - - - - 43

**Notes and News** - - - - - - - - 44

**Everybody's Page** - - - - - - - 46

**Annotations.**—Lying-In Hospitals.—Total Abstinence and Poor Relief.—Women and Faddists - - - - 4

**Round About the Asylums.**—V. - - - 48

**An Ishmaelite.** By Leslie Keith, Author of "The Chilcotes," "East and West," etc. - - - 49

**The Dangers of Celebrity** - - - - - 50

**The Editor's Letter-Box** - - - - - 51

**Amusements with Profit for all Ages.**—For Men and Women.—Children's Corner.—Puzzles, etc. - - 52

**EXTRA SUPPLEMENT.—THE NURSING MIRROR.**

En Passant.—Lewes Infirmary.—Annual Meetings.—Volunteer Nurses.—Lady Doctors.—Nursing in Canada—Lady Nurses in India.—The "Nursing Record."—The National Pension Fund v
The National Pension Fund - - - - - - vi
The Directory for Nurses, Philadelphia - - - - - vii
Everybody's Opinion.—The Hollond Institution, Nice.—Free Training in Midwifery.—A Rift in the Lute.—The British Nurses' Association - - - - - - vii
Lectures—Anecdotes of Personal Adventure—Notes and Queries—Examination Questions—Vacancies—Exchange and Mart - viii

